# Optimal treatment conditions for low-intensity pulsed ultrasound therapy for Alzheimer’s disease: applications from mice to humans

**DOI:** 10.1007/s10396-024-01461-9

**Published:** 2024-05-02

**Authors:** Tomohiko Shindo, Kumiko Eguchi, Yuto Monma, Hiroshi Kanai, Satoshi Yasuda, Hiroaki Shimokawa

**Affiliations:** 1 Department of Cardiovascular Medicine, Tohoku University Graduate School of Medicine, 1-1 Seiryo-Machiachiachi, Aoba-Kuuu, Sendai, Miyagi 980-8574 Japan; 2 Division of Biomedical Measurements and Diagnostics, Graduate School of Biomedical Engineering, Tohoku University, Sendai, Miyagi Japan; 3 Graduate School, International University of Health and Welfare, 4-3 Kozunomori, Narita, Chiba 286-8686 Japan

**Keywords:** Alzheimer’s disease, Dementia, Low-intensity pulsed ultrasound, Nitric oxide synthase, Vascular endothelial growth factor

## Abstract

**Purpose:**

We previously developed a novel therapy with low-intensity pulsed ultrasound (LIPUS) that ameliorates cognitive decline through upregulation of endothelial nitric oxide synthase (eNOS) in mouse models of Alzheimer’s disease (AD). In a randomized, double-blind, placebo-controlled pilot trial, we demonstrated that whole-brain LIPUS therapy is safe and tends to suppress the cognitive decline in early AD patients. We herein report the findings of our basic experiments that we performed for the pilot trial in order to apply whole-brain LIPUS therapy to humans, as well.

**Methods:**

First, we examined the relationship between bone density/thickness and ultrasound transmittance using human temporal bone. Next, based on the results of ultrasound transmittance, we further examined mRNA expression of VEGF, FGF2, and eNOS in response to variable ultrasound frequencies, duty cycles, and sound pressures.

**Results:**

There was a significant correlation between bone thickness and transmittance (1.0 MHz, P < 0.001), while there was no significant correlation between bone density and transmittance (1.0 MHz, P = 0.421). At a frequency of 0.5 MHz, the optimum duty cycle was considered to be up to 20%. When the tissue amplitude was in the range of 0.05–0.5 MPa, VEGF, FGF2, and eNOS were significantly upregulated by LIPUS. Thus, the conditions necessary for LIPUS therapy for the human brain were identified as sound pressure just below the probe 1.3 MPa (tissue amplitude 0.15 MPa), duty cycle 5%, and frequency 0.5 MHz.

**Conclusion:**

We successfully identified the optimal treatment conditions for LIPUS therapy for patients with AD.

## Introduction

Although the prevalence of Alzheimer’s disease (AD) has been rapidly increasing worldwide, an effective and safe disease-modifying therapy remains to be developed. Recently, endothelial dysfunction with reduced nitric oxide (NO) availability has been suggested to play an important role in the pathogenesis of AD [1]. As a novel therapeutic option for ischemic heart disease, we developed a low-intensity pulsed ultrasound (LIPUS) therapy that upregulates endogenous endothelial nitric oxide synthase (eNOS) with resultant angiogenesis and suppression of chronic inflammation [2, 3]. Moreover, we demonstrated the efficacy and safety of LIPUS therapy for several types of heart failure and cerebral infarction in animal experiments [4–8]. We also reported the results of an exploratory phase 2 clinical trial (randomized, double-blind, placebo-controlled trial) evaluating the efficacy and safety of LIPUS therapy for refractory angina [9]. Furthermore, we demonstrated in a mouse model of AD that LIPUS therapy suppresses cognitive decline and reduces amyloid-beta (Aβ) accumulation through eNOS upregulation [10]. We then conducted a randomized, double-blind, placebo-controlled pilot trial (RCT) to investigate the efficacy and safety of LIPUS therapy in patients with early AD (mild cognitive impairment due to AD and mild AD) [11]. In this pilot trial, LIPUS therapy was performed for the whole brain through the bilateral temporal bones alternatively for 1 h three times a week as one session under the special conditions that we identified [11]. LIPUS therapy was performed for a total of six sessions [11]. Among the 22 patients divided into two group in a 1:1 fashion, cognitive functions remained unchanged in the LIPUS group, while they progressively worsened at 24, 48, and 72 weeks in the placebo group [11]. Furthermore, no adverse effects were noted [11].

For this trial, we needed to solve some issues in order to apply the basic experimental data in mice to humans. First, we needed to set the ultrasonic frequency and sound pressure high enough to penetrate the thick human skull, which seemed to be 5–6 mm, while the previous evaluation was based on the thin mouse skull, which was less than 1 mm. Second, we needed to establish an ultrasonic sound field that can sufficiently cover the distribution of Aβ that accumulates not only in the human hippocampus but also in the entire brain. Third, we needed a specific ultrasonic probe for the thick human skull.

Other laboratories have also reported intracranial application of LIPUS, albeit as a different concept from our whole-brain irradiation strategy. Jeong et al. examined the acute effect of combining microbubble intravenous injection and low-intensity transcranial focused ultrasound to the right hippocampus in four patients with AD, and reported improvement in cognitive function within 24 h [12]. Popescu et al. also examined the efficacy of 2–4 weeks of transcranial ultrasound pulse stimulation (shock waves) for cortical atrophic lesions seen on magnetic resonance imaging (MRI) and reported a reduction in brain atrophy that could be associated with improved cognitive function [13]. However, these reports were based on the concept of local treatment targeting the hippocampus by using focused ultrasound, and the irradiation conditions were quite different from our whole-brain irradiation strategy. From an engineering perspective, it has been reported that a component of velocity due to vibrations induced in cells via ultrasound irradiation is the contributing parameter for the optimal conditions of LIPUS [14].

We herein report the findings of our basic experiments that we performed for the pilot trial in order to apply whole-brain LIPUS therapy to humans, as well.

## Materials and methods

The present study was performed according to the protocols approved by the Ethics Committee of Tohoku University Graduate School of Medicine (2016–1-071 and 2017–1-581). All the patients included in this study provided written informed consent. This study was conducted between 2016 and 2018. The treatment conditions for the pilot trial of LIPUS therapy (2018 ~ 2022) were determined after comprehensive evaluation, including the results of this study. This study was supported in part by grants from the Japan Agency for Medical Research and Development (16lm0103007j0005 and 17lk1403011h0001).

### Optimal sound field setting of LIPUS for intracranial therapy

**Fig. 1:**
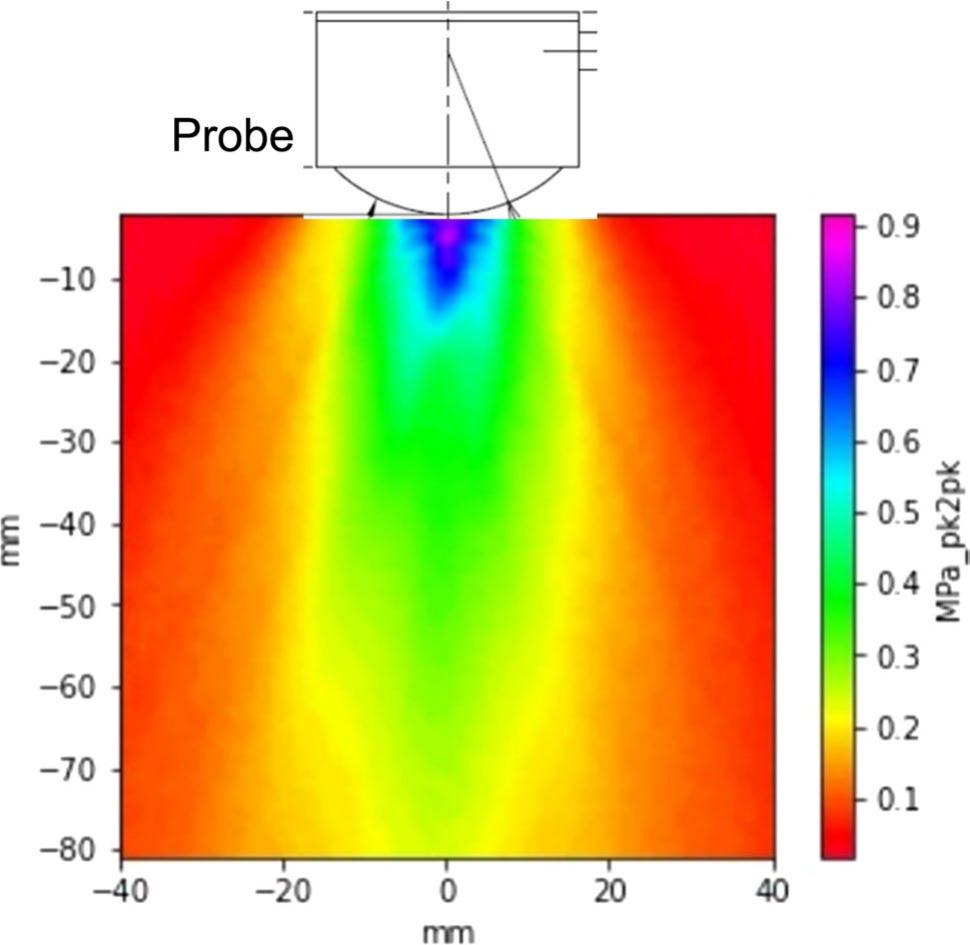
Sound pressure distribution of LIPUS therapy Sound field analysis of the ultrasound probe using the Acoustic Intensity Measurement System is shown. The energy just below the probe is the highest. We designed the ultrasound probe so that the sound pressure at a depth of 8 mm passing after the temporal bone would be within the therapeutic range (amplitude 0.5-1.0 MPa)

It has been reported that Aβ accumulates throughout the brain beyond the hippocampus as the AD progresses [15]. Thus, we aimed to apply LIPUS to the whole brain from bilateral temporal bones with the thinnest thickness in the human skull, and designed a special probe that could achieve a sufficient sound field. For LIPUS therapy, we used a multifunction generator (WF1974; NF Corporation Yokohama, Japan) with a bipolar amplifier (BA4825; NF Corporation Yokohama, Japan), and a planer ultrasound probe (Honda Electronics Co., Aichi, Japan). As an estimation of human skull volume, the radius of the curvature of the probe was set based on the Japanese skull size database (Agency of Industrial Science and Technology, Tokyo, Japan). In addition, the distance from the temporal bone to the hippocampus was determined as 75–85 mm in adult patients who underwent head MRI at Tohoku University Hospital. Thus, we designed the probe so that the sound pressure at the depth of 8 mm passed after the temporal bone from the surface to be within the therapeutic range (amplitude 0.1–0.5 MPa) (Fig. 1). The commercially available Acoustic Intensity Measurement System (Eastek Corporation, Tokyo, Japan) and PVDF Needle Hydrophone (Eastek Corporation, Tokyo, Japan) were used as sound field measurement devices. The distance from the LIPUS transducer to the hydrophone was 80 mm. In addition, for safety reasons, we set the sound pressure so as not to exceed ${I}_{{\text{spta}}}$ (spatial peak temporal average intensity) 240 mW/cm^2^ in the cranium based on the Japanese Industrial Standards.

### Relationships between bone density/thickness and ultrasound transmittance

Next, since the backbone data of LIPUS therapy are based on previous evaluation of the thin mouse skull, we needed to modify the therapeutic condition of LIPUS so that it could penetrate the human skull and exert a sufficient therapeutic effect on the brain. In this study, we used the skulls of 20 patients who underwent an autopsy at the Anatomy Department of Tohoku University Hospital in 2016. Written consent was obtained from the families of all autopsy patients who donated skulls. Since brain tissue has steady blood perfusion, sound field distribution of LIPUS was thought to be stabilized in a few minutes. Thus, by submerging the human skull in a water tank, we used a hydrophone sensor to examine how much LIPUS penetrated the skull when irradiated from the temporal bone. In addition, the temporal bone thickness $(\Delta {h}_{{\text{b}}})$ and bone density $(\uprho )$ were treated as variables that could affect the transmittance, and the correlation with the transmittance was ascertained.

The acoustic impedance of bone ${Z}_{{\text{b}}}$ is given by ${Z}_{{\text{b}}}={\rho }_{{\text{b}}}{c}_{{\text{b}}}$, and the acoustic impedance of skin and brain soft tissue (almost the same as water) ${Z}_{{\text{w}}}$ is given by ${Z}_{{\text{w}}}={\rho }_{{\text{w}}}{c}_{{\text{w}}}$. Where the sound velocity in the bone is ${c}_{{\text{b}}}$, and its density is ${\rho }_{{\text{b}}}$, the sound velocity of water in the skin and brain soft tissue is ${c}_{{\text{w}}}$, and its density is ${\rho }_{{\text{w}}}$. Transmittance of incident sound pressure from skin to bone ${T}_{12}$ and transmittance of incident sound pressure from the bone to brain soft tissue ${T}_{23}$ was given as follow:

$${T}_{12}=2{Z}_{{\text{b}}}/({Z}_{{\text{b}}}+{Z}_{{\text{w}}})$$

$${T}_{23}=2{Z}_{{\text{w}}}/({Z}_{{\text{b}}}+{Z}_{{\text{w}}})$$

On the other hand, when the propagation attenuation coefficient in the bone $\mathrm{is expressed as }{\mathrm{\alpha }}_{{\text{b}}}$, the propagation attenuation coefficient in soft tissue (water) ${\mathrm{\alpha }}_{{\text{w}}}$, skin thickness $\Delta {h}_{{\text{w}}1}$, bone thickness $\Delta {h}_{{\text{b}}}$, and brain soft tissue distance $\Delta {h}_{{\text{w}}3}=80 {\text{mm}}$, the sound pressure ${P}_{1}$ at a depth of 80 mm in the brain can be obtained as follows:

1 $$\begin{array}{*{20}c} {P_{1} = P_{0} \cdot exp( - \alpha _{{\text{w}}} \Delta h_{{{\text{w}}1}} ) \cdot T_{{12}} \cdot exp( - \alpha _{{\text{b}}} \Delta h_{{\text{b}}} ) \cdot T_{{23}} \cdot exp( - \alpha _{{\text{w}}} \Delta h_{{{\text{w}}3}} ),} \\ \end{array}$$

where the sound pressure just below the probe is ${P}_{0}$. Transmittance corresponds to ${P}_{1}/{P}_{0}$.

Transform this equation as follows:

2 $$\begin{array}{*{20}c} {ln\left( {\frac{{P_{1} }}{{P_{0} }}} \right) = \left( { - \alpha _{{\text{w}}} \Delta h_{{{\text{w}}1}} - \alpha _{{\text{b}}} \Delta h_{{\text{b}}} - \alpha _{{\text{w}}} \Delta h_{{{\text{w}}3}} } \right) + ln\left( {T_{{12}} \cdot T_{{23}} } \right)} \\ { = C_{0} - \alpha _{{\text{b}}} \Delta h_{{\text{b}}} + ln\left( {T_{{12}} \cdot T_{{23}} } \right)} \\ \end{array}$$

Here, ${C}_{0}=-{\mathrm{\alpha }}_{{\text{w}}}\Delta {h}_{{\text{w}}1}-{\mathrm{\alpha }}_{{\text{w}}}\Delta {h}_{{\text{w}}3}$ is constant in this experiment.

And, since ${Z}_{b}\gg {Z}_{w}$ holds,

$${T}_{12}=\frac{2{Z}_{b}}{{Z}_{b}+{Z}_{w}}\sim \frac{2{Z}_{b}}{{Z}_{b}}=2$$

$${T}_{23}=\frac{2{Z}_{w}}{{Z}_{b}+{Z}_{w}}\sim \frac{2{Z}_{w}}{{Z}_{b}}$$

$${T}_{12}\cdot {T}_{23}\sim \frac{4{Z}_{w}}{{Z}_{b}}=4\frac{{\rho }_{w}{c}_{w}}{{\rho }_{b}{c}_{b}}=\frac{{C}_{1}}{{\rho }_{b}{c}_{b}}$$

${C}_{1}=4{\rho }_{w}{c}_{w}$ is constant in this experiment.

Inserting these results into Eq. (2) and expressing the dependence of ${\mathrm{\alpha }}_{{\text{b}}}$ on frequency $f$ as ${\mathrm{\alpha }}_{{\text{b}}}\left(f\right)$,

3 $$\begin{array}{*{20}c} {ln\left( {\frac{{P_{1} }}{{P_{0} }}} \right)\sim C_{0} + ln(C_{1} ) - \alpha _{{\text{b}}} \left( f \right) \cdot \Delta h_{{\text{b}}} - ln\left( {\rho _{b} } \right) - ln\left( {c_{b} } \right),} \\ { = C_{2} - \alpha _{{\text{b}}} \left( f \right) \cdot \Delta h_{{\text{b}}} - ln\left( {\rho _{b} } \right) - ln\left( {c_{b} } \right),} \\ {} \\ \end{array}$$

Here, ${C}_{2}={C}_{0}+{\text{ln}}({C}_{1})$ is constant in this experiment.

Since ${\text{ln}}\left({\rho }_{b}\right)$ and ${\text{ln}}\left({c}_{b}\right)$ are logarithms, it could be assumed that they hardly change. Also, ${\mathrm{\alpha }}_{{\text{b}}}\left(f\right)$ generally increases as the frequency $f$ increases. The irradiation condition using 1.875 MHz was the standard treatment condition for the heart (angina pectoris) through the intercostal space [2, 3]. However, we found in the preliminary study that almost no transmission occurred with 1.875 MHz through the thickness of the human skull (data not shown). Thus, in order to enhance LIPUS transmittance through human skull, we examined longer wave lengths, i.e., 0.5 and 1.0 MHz.

### In vitro experiments to determine optimal irradiation conditions

In order to modify the treatment conditions for mice to those for humans, it seemed to be necessary to adjust not only the frequency but also the duty cycle and sound pressure. Thus, we next performed LIPUS irradiation experiments using cultured vascular endothelial cells and examined mRNA expression of vascular endothelial growth factor (VEGF), fibroblast growth factor 2 (FGF2), and endothelial nitric oxide synthase (eNOS). We previously reported that LIPUS upregulated not only eNOS but also cell growth factors such as VEGF and FGF2 [2, 3]. In this study, we evaluated these growth factors and confirmed the effectiveness of LIPUS. We irradiated endothelial cells with LIPUS through an agar phantom gel as previously reported [2, 3]. The culture dish for cells was filled with culture medium, and an ultrasound probe was placed to prevent air bubbles from entering between them, and irradiation was performed while keeping the probe clean. The center frequencies of the ultrasound probes used in this study were 0.5, 1.0, 1.5, and 1.875 MHz depending on the experimental system. We only used probes that met the standard of variation within ± 8% based on the ultrasonic output of 72 mW. The ultrasound output was measured using a high-performance electronic balance-type low-ultrasound-power measuring device (UPM-DT-1E; Eastek Corporation, Tokyo, Japan). The probe shape was a circular planar element (diameter 26 mm, 28 mm). The attenuation coefficient ${\mathrm{\alpha }}_{{\text{w}}}$ of the phantom gel was almost comparable to that of living cells (e.g., muscles, fat, and blood). Human umbilical vein endothelial cells (HUVEC) from a single donor (Lonza, Basel, Switzerland) were cultured in a complete endothelial medium (EGM-2 BulletKit, Lonza). The cells were used at passages three to five and were maintained in EGM-2. Twenty-four hours before LIPUS treatment, the cells (1 × 10^5^) were re-suspended in a 2-ml tube with EGM (Lonza). They were exposed to LIPUS under the following irradiation conditions (frequency: 0.5, 1.0, and 1.875 MHz; duty cycle: 1, 5, 10, 20, and 40%; amplitude: 0.05, 0.1, 0.15, 0.25, 0.5, 1.08, and 2.2 MPa) for 20 min (n = 12 each). Since heat generation on the element surface cannot be ignored at a duty ratio of 40% or more, we only examined the duty ratio under conditions of 1, 20, and 40%. Regarding sound pressure, referring to our previous reports, it was thought that sound pressure over 2.2 MPa is highly cytotoxic [2, 3]. The pulse repetition rate (*PRT*) was fixed at 320 μs to comply with previous reports [2, 3] and safety standards [16]. After irradiation, the cells were stored for 6 h in the same medium before RNA extraction. mRNA was extracted using the RNeasy Plus Mini kit (QIAGEN). mRNA (600 ng) was reverse- transcribed using a QuantiTect Reverse Transcription kit (QIAGEN). Real-time PCR was performed using the Real-Time Detection System (Bio-Rad Laboratories). cDNA was synthesized by using PrimeScript RT Master Mix (Takara Bio Inc., Tokyo, Japan). The primer sequences were as follows: VEGFA, (Forward) 5’-GAGCCTTGCCTTGCTGCTCTA-3’ and (Reverse) 5’-CACCAGGGTCTCGATTGGATG-3’; FGF2, (Forward) 5′- GTGTGCTAACCGTTACCTGGCTATG-3′ and (Reverse) 5′- CCAGTTCGTTTCAGTGCCACA-3′; eNOS, (Forward) 5’-AAAGACAAGGCAGCAGTGGAAAT-3’ and (Reverse) 5’-TCCACGATGGTGACTTTGGCTA-3’; GAPDH, (Forward) 5’-GCACCGTCAAGGCTGAGAAC-3’ and (Reverse) 5’-TGGTGAAGACGCCAGTGGA-3’, all of which were designed with the Perfect Real Time Support System (Takara Bio Inc.). For the final validation, the irradiation conditions considered to be the optimal conditions for LIPUS treatment were applied to cerebral vascular endothelial cells (Lonza), and VEGF mRNA expression was also evaluated.

### Statistical analysis

Results are shown as mean ± standard deviation (SD) for all experiments. We used Student’s t-test followed by Bonferroni multiple comparisons and 2-way ANOVA with Turkey’s HSD multiple comparison test to compare mean values (GraphPad Prism Software, San Diego, CA). Differences with a P-value < 0.05 were considered to be statistically significant.

## Results

### Relationships between bone density/thickness and ultrasound transmittance

**Fig. 2:**
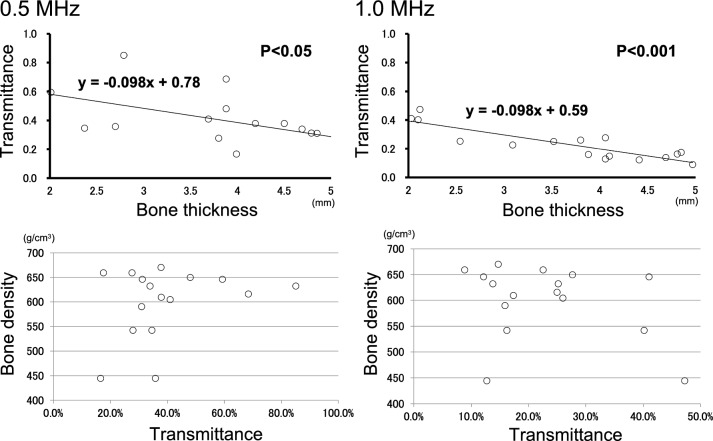
Correlation diagram among LIPUS transmittance, bone thickness, and bone density Although there was no significant correlation between bone density and transmittance (1.0 MHz, P = 0.421), there was a significant correlation between bone thickness and transmittance (1.0 MHz, P < 0.001). The transmittance was higher at lower frequencies (0.5 MHz: y = -0.098x + 0.78, expected transmittance at 2 cm under skull 58.4%; 1.0 MHz: y = -0.098x + 0.59, expected transmittance at 2 cm under skull 39.4%)

Using human temporal bone, we examined the relationship between bone density/thickness and ultrasound transmittance. The sound field shown in Fig. 1 is the plotted data for an excitation frequency of 0.5 MHz using a probe with a center frequency of 0.5 MHz. Figure 1 shows the reference measurement values of ultrasonic waves, where the energy just below the probe is the highest. There was a significant correlation between bone thickness and transmittance (1.0 MHz, P < 0.001) (Fig. 2). Also, the transmittance was higher at lower frequencies (0.5 MHz: y = -0.098x + 0.78, expected transmittance at 2 cm under skull 58.4%; 1.0 MHz: y = -0.098x + 0.59, expected transmittance at 2 cm under skull 39.4%) (Fig. 2). In this verification using a human skull, we found that the transmittance of 0.5 MHz was higher than that of 1.0 MHz at any thickness within the skull thickness range of 2–5 mm (Fig. 2). Based on these results, we modified the frequency from 1.875 MHz for the heart through the intercostal space [2, 3] to 0.5 MHz for the brain through the human skull.

### In vitro experiments to determine optimal irradiation conditions

**Fig. 3:**
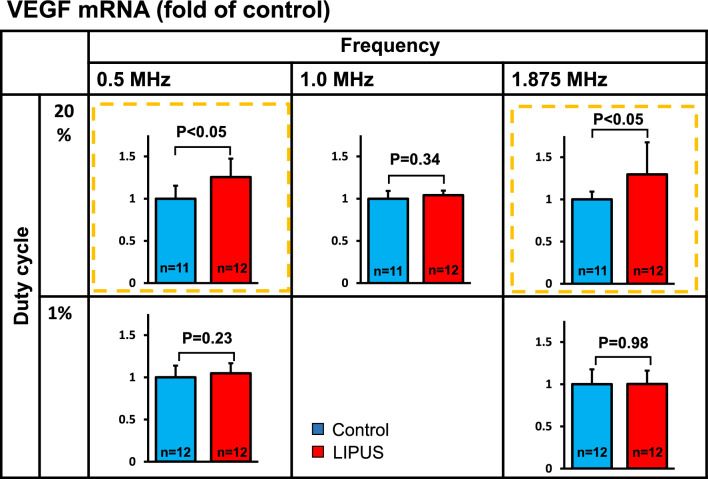
VEGF expression in response to LIPUS for each duty cycle and frequency In an in vitro study, the frequency was first fixed at 0.5 MHz, and the duty cycle was changed to 1, 20, and 40% to examine changes in VEGF mRNA expression after irradiation with LIPUS. When the duty cycle was fixed at 20%, LIPUS-induced mRNA expression of VEGF was significantly increased at 0.5 MHz and 1.875 MHz (P < 0. 05)

**Fig. 4:**
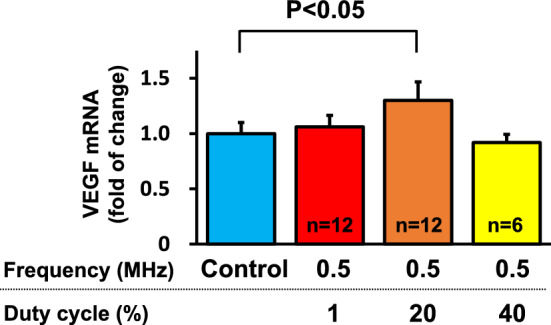
LIPUS-induced VEGF expression for each duty cycle Next, we fixed the frequency to 0.5 MHz and evaluated the duty cycle by changing it to 1, 20, and 40%. mRNA expression of VEGF in response to LIPUS was significantly increased at a duty cycle of 20% (P < 0.05), but not at 1 or 40%

**Fig. 5:**
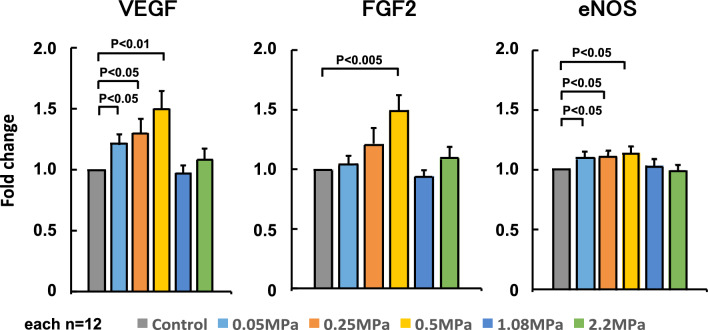
Effects of different sound pressure on LIPUS-induced mRNA expression of VEGF, FGF2, and eNOS We examined the effects of different sound pressures (amplitudes) at a fixed frequency of 0.5 MHz and a duty cycle of 20% on mRNA expression of VEGF, FGF-2, and eNOS. VEGF and eNOS expression were significantly increased in the range of 0.05-0.5 MPa, as well as expression of FGF-2 at 0.5 MPa, but no significant upregulation was noted at the greater pressures

**Fig. 6:**
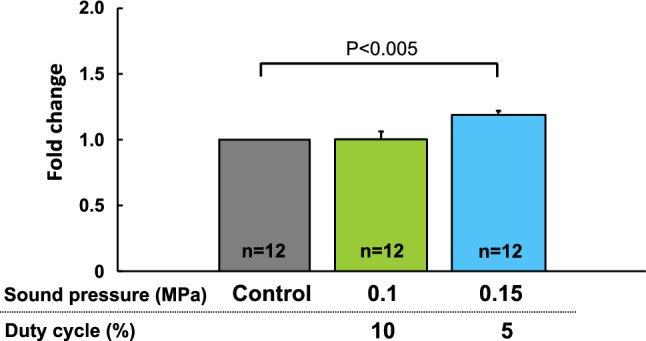
Comparison of VEGF expression levels under two treatment conditions We compared VEGF mRNA expression under two treatment conditions: (1) sound pressure just below the probe of 1.3 MPa (tissue amplitude 0.15 MPa) with each duty cycle 5%; (2) sound pressure directly below the probe 0.9 MPa (tissue amplitude 0.1 MPa) with each duty cycle 10%. VEGF mRNA was significantly upregulated under condition 1 as compared with condition 2

Using human umbilical vein endothelial cells in vitro experiments, the frequency was first fixed at 0.5 MHz, and the duty cycle was changed to 1, 20, and 40% to examine changes in VEGF mRNA expression after LIPUS irradiation. When the duty cycle was fixed at 20%, significant LIPUS-induced mRNA expression of VEGF was observed at 0.5 and 1.875 MHz (P < 0. 05), whereas the expression did not increase at 1% duty cycle **(**Fig. 3). Thus, when the frequency was fixed at 0.5 MHz, VEGF mRNA expression significantly increased at a duty cycle of 20% (P < 0. 05) (Fig. 4). These results indicated that the optimal frequency was 0.5 MHz and the optimum duty cycle was up to 20% (10% from the right and left probes). We used the frequency of 1.875 MHz in our previous experiment in mice with a thin skull [10]. Next, we fixed the irradiation condition at a frequency of 0.5 MHz and a duty cycle of 20%, and the range of sound pressure (amplitude) was changed to 0.05–2.2 MPa, which is a sound pressure that can be set with a convex transducer. Under these conditions, we examined mRNA expressions of VEGF, FGF2, and eNOS. As shown in Fig. 5, VEGF and eNOS expression was significantly upregulated in the range of 0.05–0.5 MPa, and FGF expression at 0.5 MPa, without any effects at sound pressures greater than 1.08 MPa. As we reported previously [2], when the sound pressure is 1.08 MPa or higher, it is highly cytotoxic and may cancel its effectiveness. Since effectiveness is lost for each growth factor when the sound pressure consistently exceeds 1.08 MPa, we decided that conditions above this sound pressure should not be used. Similarly, when the duty cycle exceeds 40%, cytotoxicity is thought to increase [2], which suggests that the optimal tissue amplitude is 0.05–0.5 MPa. In order to achieve this sound pressure in the human brain, it was estimated that the sound pressure just under the probe needed to be in the range of 0.5–1.5 MPa. We ultimately set the treatment conditions to be under the safety standard (Ispta not to exceed 240 mW/cm^2^), and above the efficacy standard (sound pressure should be more than 0.5 MPa directly below the probe). Therefore, the following two treatment conditions were compared for VEGF mRNA expression using cerebrovascular endothelial cells: (1) sound pressure just below the probe of 1.3 MPa (tissue amplitude 0.15 MPa) and each duty cycle 5%; (2) sound pressure just below the probe of 0.9 MPa (tissue amplitude 0.1 MPa) and each duty cycle 10%. In the final verification experiment, cerebrovascular endothelial cells were used in order to confirm actual reactivity in the irradiated brain. In this comparative experiment, VEGF mRNA expression was significantly upregulated under condition 1 as compared with condition 2 (Fig. 6).

## Discussion

In the present study, we aimed to determine the optimal treatment conditions for LIPUS therapy in humans after previous experiements in a mouse model of AD. We were able to identify the treatment conditions for human brain, including sound pressure just below the probe of 1.3 MPa (tissue amplitude 0.15 MPa), each duty cycle 5%, and frequency 0.5 MHz.

In our previous basic experiments using animal models of heart disease, we performed LIPUS therapy under the following conditions: center frequency 1.875 MHz, pulse repetition frequency 2.74 kHz, number of cycles 32 (17 $\mathrm{\mu s}$ burst length, i.e., duty cycle = 4.7%), ${I}_{{\text{spta}}}$ (spatial peak temporal average intensity) 117–162 mW/cm^2^, and sound pressure 0.25 MPa [2–8, 10]. We demonstrated that this treatment condition physically stimulated the caveolae on vascular endothelial cell membranes and induced a series of intracellular signals that promote cell proliferation and tissue repair via eNOS [17]. In the previous animal experiments in mice, the thickness of the mouse skull was less than 1 mm, so the attenuation of ultrasound transmission was negligible. However, it was difficult to ignore the attenuation when the same condition was applied to the human skull. In the present study, the thinnest temporal bone in the human skull was about 3–5 mm, and at 0.5 MHz, it was possible to deliver ultrasonic waves to the cranium with an attenuation rate of 30–40%. In addition, since the accumulation of Aβ in AD spreads to the entire brain as it progresses, we designed a diffusion-type therapeutic beam that ensures safety in compliance with Japanese Industrial Standards. We confirmed this ultrasonic sound field (Fig. 1**)**. In the clinical trial, we designed it so as to produce sound pressure of 0.15 to 0.19 MPa in the deepest part of the hippocampus (approximately 8 cm deep), and sound pressure just below the skull needed to be 1.0 to 1.5 MPa or less to ensure safety [11]. The cone angle of the diffusive probe was set according to the size of the average elderly Japanese skull.

The next issue was the effect of bone properties such as thickness and density on sound pressure transmittance. In elderly patients, in particular, we thought that bone density could not be ignored, but no significant correlation was found between bone density and transmittance in this study. Interestingly, the scatterplot linear functions of bone thickness and transmittance coincidentally showed a negative correlation with exactly the same slope (-0.098) at 0.5 MHz and 1.0 MHz. The original LIPUS therapy developed for heart disease was set at a frequency of 1.875 MHz based on the standard for diagnostic ultrasound devices for the heart. However, we needed to modify the frequency to lower than 10 MHz, so that LIPUS could sufficiently penetrate the human skull. Investigation using vascular endothelial cells showed that LIPUS-induced VEGF mRNA expression could be reproduced even at 0.5 MHz. However, we were unable to identify why VEGF upregulation was not observed under a frequency of 1.0 MHz and duty cycle of 20% or a frequency of 0.5 MHz and duty cycle of 1%. We then found that when fixing the frequency at 0.5 MHz, the optimal duty cycle was up to 20%. Furthermore, by evaluating mRNA expression of VEGF, FGF2, and eNOS, we found that the optimal sound pressure was 0.05 to 0.5 MPa. Based on these results, the optimal treatment conditions were ultimately narrowed down to two conditions from the upper limit of the safety criteria, and the expression effect of VEGF was retested.

The present results were applied to the pilot trial to address the efficacy and safety of LIPUS therapy for patients with early AD with promising results [11]. In this pilot trial, eligibility criteria included patients with a Clinical Dementia Rating (CDR) global score of 0.5–1.0 and a Japanese Mini Mental State Examination (MMSE-J) score more than 20 [11]. Either the LIPUS treatment or the placebo procedure was performed in outpatients for 18 months at 3-month intervals for a total of six sessions (18 treatments). In the placebo group, changes in ADAS-J cog, the primary endpoint, worsened over time at 24, 48, and 72 weeks, whereas no changes were noted in the LIPUS-treated group [11].

Several limitations should be mentioned for the present study. First, regarding sound field measurements, it is assumed that the influence of propagation attenuation was smaller compared to the biological tissue, because we used water in this study. Since it is difficult to accurately predict the propagation attenuation in actual brain tissue, we used water as an alternative to brain tissue that is rich in blood flow. We also verified that the risk is within an acceptable range, e.g., the sound pressure does not become too high. Second, it has been reported that the cancellous and cortical bone volume ratio is related to the propagation of ultrasound [18]. In this study, we did not measure the cancellous and cortical bone volume ratio, which may have affected ultrasound transmittance. This point remains to be examined in a future study. Third, in the present study, although we used a human skull, irregular reflection was difficult to simulate completely. Thus, it was difficult to reliably verify the safety of intracranial ultrasound diffuse reflection. Fourth, we used cultured vascular endothelial cells to determine therapeutic ultrasound conditions, but it may have been necessary to examine its efficacy in vivo using large animals. Fifth, although the present study was performed on the premise of output within the safety standards of the Japanese Industrial Standards, it may have been necessary to verify more effective output conditions than the present standard.

## Conclusion

We performed basic experiments for the "whole-brain irradiation" concept of human intracranial LIPUS therapy, and we successfully identified the optimal treatment conditions in humans. We plan to employ the same LIPUS therapy conditions in the next pivotal clinical trial, as well.

## Data Availability

Raw data were generated at Tohoku University. Derived data supporting the findings of this study are available from the corresponding author H.S. on request.
